# Multi-way radial consistency pre-training for event based optical flow

**DOI:** 10.3389/fnbot.2026.1884626

**Published:** 2026-07-16

**Authors:** An Feng, Tao Wenyin

**Affiliations:** 1 Department of Biotechnology, Suzhou Industrial Park Institute of Services Outsourcing, Suzhou, Jiangsu, China; 2 College of Computer Science and Technology, Nanjing University of Aeronautics and Astronautics, Nanjing, Jiangsu, China

**Keywords:** event camera, multi-way self-supervision, optical flow, polar tessellation, radial consistency

## Abstract

Optical flow estimation is a low-level module in computer vision, widely used in tasks such as visual odometry, autonomous driving, high dynamic range (HDR) imaging, and action recognition. Existing event-based optical flow estimation approaches suffer from scarcity of dense real-world datasets, while some unsupervised frameworks have reduced reliance on large-scale datasets by forward-backward consistency loss, they primarily exploit a 1D temporal reversal, while largely ignoring the rotational and scaling motions ubiquitous in robotics and automotive scenes. This work introduces radial consistency, a self-supervised pre-training framework that maps the event stream to log-polar coordinates and tessellates the spatial domain into *K* radial rings and *L* angular sectors, a shared encoder-decoder to predict four complementary flow fields whose cyclic sum is driven to zero, yielding a closed-loop constraint that generalizes the classical forward-backward check to 360° within a sector. Our *core contribution*, the radial consistency loss, is completely label-free, together with auxiliary terms, enabling self-supervised pre-training on large-scale event data. We optionally apply supervised fine-tuning on small labeled sets to adapt to specific domains, achieving competitive accuracy with fully supervised methods. Validation experiments on Multi Vehicle Stereo Event Camera (MVSEC) dataset demonstrate strong performance: our method achieves 0.67 EPE averaged across all sequences, surpassing E-RAFT (0.89 EPE) and EV-FlowNet (1.10 EPE), without any additional data. On the rotation-heavy indoor_flying3 sequence specifically, we achieve 0.93 EPE (fine-tuned) and 1.49 EPE (self-supervised only) vs. E-RAFT 1.66. We also improve upon E-RAFT in computational efficiency [55 frames per second (FPS) and 26 giga floating-point operations (GFLOPs) vs. 42 FPS and 38 GFLOPs], while requiring only minimal supervised fine-tuning.

## 1 Introduction

Optical flow estimation aims to capture the pixel-level motion between consecutive frames. It is a fundamental module in many computer vision tasks, including visual odometry, autonomous driving, HDR imaging, and action recognition. Traditional methods typically treat the estimation of optical flow as a variational energy minimization problem in which a data fidelity term and a spatial smoothness term are jointly optimized (Horn and Schunck, 1981). While recent deep learning approaches directly regress flow fields from images (Dosovitskiy et al., 2015; Ilg et al., 2017; Sun et al., 2018; Teed and Deng, 2020; Jiang et al., 2021; Meister et al., 2018).

However, frame-based sensors are intrinsically constrained by millisecond exposure times and limited dynamic range, inevitably introducing motion blur and over/under exposure in high speed or low light scenes. Event cameras, biologically inspired vision sensors, output asynchronous event streams with microsecond temporal precision and logarithmic intensity encoding, offering >120 dB dynamic range and ultra low power consumption. They are regarded as a highly suitable choice for addressing these issues in high-speed or high-dynamic-range scenarios.

Just following the frame-based ideas, the community tried to accumulate events into voxel or pseudo images, and then applying ConvNets inherited from classical flow. These pipelines do deliver impressive numbers when tested on the same synthetic dataset they were tuned on, but often suffer from severe performance degradation when the camera undergoes significant rotation or zoom, or when illumination changes abruptly. Two root causes are (i) the absence of large scale real data with dense ground truth and (ii) the loss of the event sensor's native temporal continuity when one squeezes an asynchronous stream into a 4-channel tensor.

In this paper we keep the tensor, but we stop treating it as an ordinary image. Instead, we regard it as an angular slice of an omnidirectional motion field. We tessellate the event volume in log-polar coordinates, a shared encoder-decoder architecture is constrained to predict four complementary flows {**f**_→_, **f**_←_, **f**_*s*_, **f**_*r*_} whose cyclic sum converges to zero within each polar sector (*k, l*), i.e., $\underset{i}{\sum }{\text{f}}_{i}^{\left(k,l\right)}=\text{0}$. Training employs our radial consistency loss as the primary geometric supervisory signal, complemented by auxiliary self-supervised terms for robust convergence.

We embed this radial consistency loss inside a unsupervised framework that first pre-trains on unlabeled data and then refines on a handful of labeled sequences. A Swin Transformer with shifted temporal windows supplies global context without ever building an explicit cost volume. The resulting network runs at 55 FPS on a single RTX-3060, achieves an EPE of 0.67 on average in the MVSEC sequences and 0.63 in DSEC Flow (DSEC-Flow) dataset.

## 2 Related work

We split the discussion into four themes: (i) classical and deep frame-based optical flow networks, (ii) learning-based event flow methods that still rely on intensity images or dense labels, (iii) self-supervised event flow works that use simple forward-backward consistency, and geometric constraints that exploit radial motion but have never been cast as a trainable loss. To the best of our knowledge no previous work has turned a polar tessellation of the event voxel into a differentiable cycle consistency term.

### 2.1 Frame-based optical flow

Traditional frame-based optical flow methods formulate the problem as a variational energy minimization task (Horn and Schunck, 1981). While FlowNet (Dosovitskiy et al., 2015) pioneered the deep learning era with convolutional neural network (CNN) encoder-decoder structure, followed by FlowNet2 (Ilg et al., 2017), PWC-Net (Sun et al., 2018), RAFT (Teed and Deng, 2020), and unsupervised approaches such as UnFlow (Meister et al., 2018). Although these networks have steadily improved accuracy and efficiency, they remain constrained to the quality of the frame-based inputs.

### 2.2 Event-based optical flow

Early event-based approaches maximize image contrast by warping events into sharp frames (Gallego et al., 2020). Deep learning models such as EV-FlowNet (Zhu et al., 2018b) encode events as four-channel pseudo images and regress flow with convolutional networks. More recent work incorporates depth, ego motion, and iterative refinement, cost volume, attention, state space models (Zhu et al., 2019; Ye et al., 2018; Gehrig et al., 2021b; Liu et al., 2023; Xu et al., 2026; Humais et al., 2025). While these approaches have pursued higher accuracy through increasingly intricate architectures, they inevitably sacrifice efficiency and still fall short of addressing fundamental issues such as heavy dataset dependence and poor generalization to real-world scenarios.

### 2.3 Bidirectional loss: from symmetry to asymmetry

UnFlow first replaced the traditional forward-only photometric term with a symmetric bidirectional census loss for frames, assuming equal penalties for forward and backward warps. Subsequent frame-based works soften this axiom by re-weighting the two terms with an occlusion mask (Wang et al., 2018; Janai et al., 2018), yet the sum of penalties remains balanced. When the idea was ported to event cameras, BAT (Xu et al., 2026) computed a bidirectional correlation volume but still enforced equal weights; the cavity problem caused by reversed polarity time order is mentioned but not solved. Shu et al. (2020) employs a learnable gate to down-weight the backward photometric error at occluded pixels, but the overall bidirectional penalty remains symmetric (gate weights sum to one) and is designed for dense frame inputs. Very recent state space models (Humais et al., 2025; Ding et al., 2022) continue to adopt symmetric photometric losses for events, leaving the sparse cavity issue intact.

To clarify the distinction from classical forward-backward consistency: conventional methods enforce **u**_*t*→*t*+δ_+**u**_*t*+δ → *t*_ = **0** only along the temporal dimension (1D), which constrains translational motion but imposes no restriction on rotation angles. In rectangular coordinates, while vectors can transform at any angle, this freedom is precisely the limitation, it provides no geometric constraint on rotational or zoom motions. Radial consistency addresses this by tessellating the event volume into log-polar sectors where rotation becomes translational along θ and scaling becomes translational along *r*. By enforcing cyclic closure over 360°, we explicitly constrain the angular degrees of freedom that forward-backward checks ignore.

### 2.4 Polar-cycle constraints: from hand-crafted prior to differentiable loss

Radial motion models have appeared in ego rotation detection (Werpup and Pajdla, 2001; Geyer et al., 2005), cylindrical panorama stitching (Szeliski, 1996), and simultaneous localization and mapping (SLAM) systems that assume pure rotation (Mueggler et al., 2016). These methods either hard code ω × *r* as a residual or use it to reject outliers; they do not partition the image into learnable polar bins, nor do they embed the constraint inside an end-to-end network loss. We bridge this gap by tessellating the event voxel into polar sectors, compute block-wise cycle errors, and back-propagate through the tessellation, yielding a self-supervisory signal that is explicitly tailored to the rotational geometry of event streams.

Closest to ours is the bidirectional reliability weighting of BAT, which lets the lower error direction dominate. We start from the same asymmetry principle, but tessellate the event voxel into *K*×*L* polar sectors, re-weight outer rings by an exponentially decaying radial confidence, and back-propagate only through non-empty bins. The resulting radial consistency loss is therefore the first differentiable polar-tessellated cycle loss that automatically masks empty event bins within an unsupervised event-only framework.

## 3 Method

We first formalize the event generation model, the polar cycle prior and the event voxel representation (Section 3.1), and then give the network architecture, a lightweight encoder-decoder with shifted window Swin attention (Section 3.2). The core contribution is Section 3.4, a composite loss that (i) breaks the symmetry of classical bidirectional losses to avoid “cavity” hallucination and (ii) embeds rotation/zoom geometry into a differentiable polar-tessellated cycle term. Inference details are summarized in Section 3.5.

### 3.1 Event generation & polar cycle prior & Representation

#### 3.1.1 Event generation

An event camera continuously monitors the temporal derivative of log intensity for every pixel, and an event is triggered whenever this derivative exceeds a preset threshold *T*, as expressed in Equation 1,


$$\begin{array}{l} \log\left(I_{t+1}\right)-\log\left(I_{t}\right)\geq T. \end{array}$$ (1)

The sensor's output is a discrete stream of events, each described by Equation 2, where *x, y* denote pixel coordinates, *t* the microsecond timestamp, and *p* the polarity indicating the direction of intensity change.


$$\begin{array}{l} e_{i}\left(t\right)=\left(x_{i},y_{i},t,p_{i}\right). \end{array}$$ (2)

**Figure 1:**
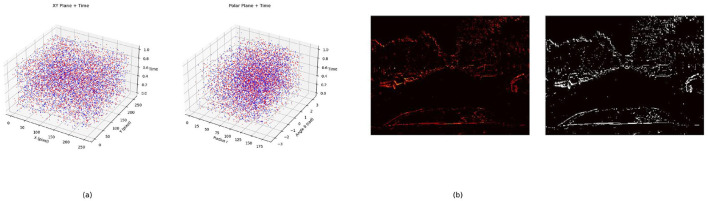
Raw event stream (Cartesian, polar) and representation (count, timestamp). **(a)** Cartesian vs. polar coordinate transformation. **(b)** Event count and timestamp representation.

To explicitly model rotation and zoom motions, we convert the event stream from Cartesian (*x, y*) coordinates to polar (*r*, θ) coordinates. The transformation is defined as Equation 3. After converting, the event stream keeps dimension of 4*n*, just like Figure 1a.


$$\begin{array}{l} \begin{array}{l} \;r_{i}=||\left(x_{i}-c_{x},y_{i}-c_{y}\right)|{|}_{2},\\ \;\theta_{i}=\arctan2\left(y_{i}-c_{y},x_{i}-c_{x}\right)\in \left[-\pi ,\pi \right],\\ e_{i}^{\text{polar}}=\left(r_{i},\theta_{i},t,p_{i}\right). \end{array} \end{array}$$ (3)

#### 3.1.2 Polar tessellation

Following the coordinate transformation in Equation 3, we apply *log-polar tessellation* to non-uniformly discretize the event stream into *K*×*L* spatial sectors. This tessellation allocates finer spatial resolution to the central region where events are dense, while using coarser bins for the periphery, matching the foveated distribution of motion information in event data.

##### Logarithmic radial mapping

To achieve exponentially increasing ring widths, we map the radial coordinate *r* to a logarithmic scale, as expressed in Equation 4.


$$\begin{array}{l} \rho =\log\left(1+\frac{r}{r_{0}}\right), \end{array}$$ (4)

where *r*_0_ is a reference radius (set to *r*_0_ = 5 px for images 256 × 256) that prevents numerical instability near the origin. This logarithmic compression ensures that inner rings capture fine-grained motions while outer rings accommodate larger displacements with proportionally lower resolution.

##### Discrete tessellation

We uniformly partition the log-radius ρ into *K* bins and the angular coordinate θ into *L* sectors. The bin boundaries are defined as Equation 5:


$$\begin{array}{l} \rho_{k}=\log\left(1+\frac{r_{\min}}{r_{0}}\right)+\frac{k}{K}\log\left(\frac{r_{\max}+r_{0}}{r_{\min}+r_{0}}\right),\;\theta_{l}=-\pi +\frac{2\pi l}{L}, \end{array}$$ (5)

where the ring *k* covers ρ∈[ρ_*k*_, ρ_*k*+1_) and the sector *l* covers θ∈[θ_*l*_, θ_*l*+1_). where *r*_min_ = 0 and *r*_max_ = 128 px for center-cropped images 256 × 256. Each sector (*k, l*) thus corresponds to a specific region in the log-polar space, and the event count (or accumulated polarity) within each sector forms the spatial foundation of our voxel representation.

Notably, classical forward-backward consistency (Meister et al., 2018) assumes optical flow arises from pure translational camera motion, where reversing the temporal direction simply inverts the displacement vector. However, event cameras frequently undergo rotational motion (e.g., drone yaw, vehicle turning), which induces *non-uniform* flow fields where displacement magnitudes scale with radius (|**f**|∝*r*). Under such SE(2) transformations, the naive inverse flow **f**_*t*+1 → *t*_ = −**f**_*t*→*t*+1_ violates the actual rotational geometry, as pixels at different radii traverse different arc lengths (Supplementary Figure S1). By tessellating the event volume into log-polar sectors, we transform rotation and scaling into linear motions along the angular and radial axes, respectively. This enables us to decompose the motion field into four complementary components (forward, backward, scaling, rotation) and enforce *multi-way cyclic consistency* within each sector, a constraint that generalizes the classical forward-backward check to the full 360° motion space.

##### Completeness of the four-flow basis

In the log-polar event voxel, pixel motion is governed by the planar similarity group Sim(2), which has four generators: *x*-translation, *y*-translation, rotation, and isotropic scaling. Under the log-polar mapping (*x, y*)↦(ρ = log*r*, θ) with the optical center as the origin, these generators project onto only two independent spatial axes as expressed in Equation 6.


$$\dot{\rho }=\frac{x\dot{x}+y\dot{y}}{r^{2}},\;\dot{\theta }=\frac{x\dot{y}-y\dot{x}}{r^{2}}.$$ (6)

Rotation becomes pure angular motion ($\dot{\theta }$), scaling becomes pure radial motion ($\dot{\rho }$), and translations decompose into coupled radial and angular components. Therefore, any instantaneous 2-D image motion is fully described by the tuple (Δρ, Δθ). To embed this geometry into a learnable cycle-consistency framework, we introduce four complementary decoder heads that predict discretized versions of these motion components:

- Forward flow **f**_→_: temporal displacement from the current voxel to the future voxel;
- Backward flow **f**_←_: temporal displacement from the future voxel back to the current voxel;
- Scaling flow **f**_*s*_: displacement along the log-radius axis ρ, modeling zoom-in/zoom-out;
- Rotation flow **f**_*r*_: displacement along the angular axis θ, modeling camera yaw or pan.

These four components span the independent directions of motion in the polar spatio-temporal volume: two spatial axes (radial and angular) and two temporal orientations (past and future). They constitute an *over-complete basis* for the 2-D motion field: the redundancy (four vectors for two degrees of freedom) is precisely what enables the cyclic closure constraint **f**_→_+**f**_*s*_+**f**_*r*_+**f**_←_≈**0** to serve as a self-supervised regularizer without ground-truth labels.

#### 3.1.3 Event representation

Formally, the raw output of an event camera was recorded as a set with dimension of 4*n*. However, standard CNNs expect dense tensor-like inputs, which requires the stream must be converted into an image-like representation. EV-FlowNet assumes grayscale frames *I*_0_ and *I*_1_ are available at times *t*_0_ and *t*_1_, and accumulates every event falling between them into a 4-channel pseudo image: channel 0 and 1 store the number of positive and negative events per pixel, while channel 2 and channel 3 for the timestamp of the latest positive and negative event per pixel. The right-hand side of Figure 1 shows the event counts channels and the latest timestamp channels.

When dense or large displacement motion occurs, both the event count and the last timestamp representations inevitably overwrite the same pixel, causing motion information to be lost. We therefore adopt the discrete event volume introduced by Zhu et al. (2019), which we extend to polar coordinates by replacing (*x, y*) with (*r*, θ). The adapted event voxel is expressed as: Equation 7:


$$\begin{array}{l} \begin{array}{l} V=\underset{i}{\sum }p_{i}k_{b}\left(r-r_{i}\right)k_{b}\left(\theta -\theta_{i}\right)k_{b}\left(t-t_{i}^{*}\right),\\ t_{i}^{*}=\left(B-1\right)\frac{t_{i}-t_{0}}{t_{N-1}-t_{0}}. \end{array} \end{array}$$ (7)

*N* is the total number of events in the time window; *p*_*i*_ is polarity of event; (*r*, θ) are spatial-temporal polar coordinates of event; *t*_0_ is start time of the event voxel; $t_{i}^{*}$ is the sampled time; *V*∈[*K, L, B*] is the voxel through bilinearly sampling. Equation 7 discretizes the timestamp into *B* temporal bins. Consequently, the voxel at any moment *t* is obtained by bilinearly sampling $t_{0}^{*}\sim t_{N-1}^{*}$ on these bins. Compared with the count and timestamp maps, the event voxel *V*(*x, y, t*) keeps the temporal distribution of events while still being an ordinary *B*-channel tensor that can be fed directly to an ANN for feature extraction and optical flow estimation.

### 3.2 Network architecture

Traditional bidirectional methods make use of a forward event voxel *V*_*f*_(*t*) and a backward event voxel *V*_*b*_(*t*); the former is discretely sampled via Equation 7 from raw events stream, the latter is from reversed time events. Both voxels pass into the network and calculate the bidirectional loss according to the forward-backward optical flow, to guide the unsupervised training progress.

We adopt the forward-backward consistency checking, but we go further by transferring the events stream from Cartesian to polar, and calculate the loss by four decoders. The whole network includes three parts: event representation in Section 3.1, feature enhancement in Section 3.3 and radial consistency loss in Section 3.4.

**Figure 2:**
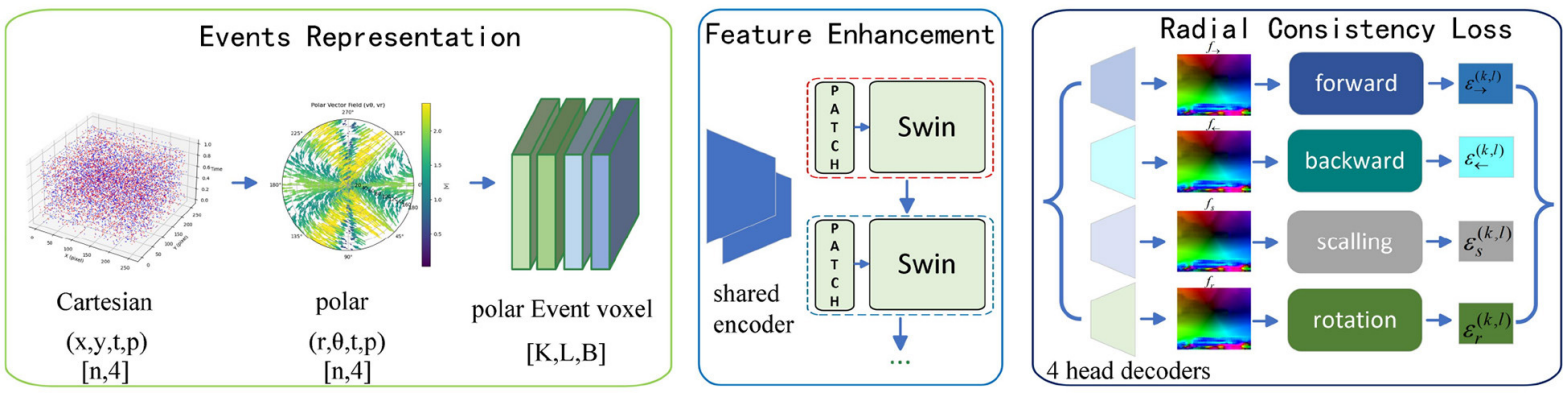
Network structure. **(Left)** Cartesian (*x, y, t, p*) to polar (*r*, θ, *t, p*) to polar Event voxel [*K, L, B*]. **(Middle)** Shared encoder with Swin Transformer blocks. **(Right)** 4 head decoders: (1) forward flow **f**_→_ (temporal progression), (2) backward flow **f**_←_ (temporal reversal), (3) scaling flow **f**_*s*_ (radial zoom-in/out), and (4) rotation flow **f**_*r*_ (angular yaw/pan). The four flows are constrained by cyclic closure $\underset{i}{\sum }{\text{f}}_{i}^{\left(k,l\right)}\approx \text{0}$ within each polar sector (*k, l*).

We adopt a lightweight encoder-decoder backbone with shared representation learning. The shared encoder ingests a single log-polar event voxel (*K, L, B*) and outputs a common feature map (*K, L, C*). Four parallel decoder heads then regress four complementary flows (forward, backward, scaling, rotation) in one forward pass, each with dimension of (*K, L*, 2). Each head is a lightweight Conv-gated recurrent unit (GRU) that refines the flow field iteratively. The overall network architecture is illustrated in Figure 2.

#### 3.2.1 Physical interpretation of the four flows

To aid readers unfamiliar with event-camera geometry, we provide an intuitive physical analogy: imagine a drone performing *forward translation* while simultaneously *yawing to the left* and *descending toward the ground*. In the log-polar event voxel, this composite motion decomposes into four independent components:

- Forward flow (**f**_→_): captures the temporal progression from the current event voxel to the next, analogous to the drone moving forward in time;
- Backward flow (**f**_←_): represents the inverse temporal check, as if the drone were to reverse its trajectory and return to the starting point;
- Scaling flow (**f**_*s*_): encodes radial expansion or contraction (zoom-in/zoom-out), corresponding to the drone descending (objects appear larger) or ascending (objects appear smaller);
- Rotation flow (**f**_*r*_): encodes angular motion around the optical axis, corresponding to the drone's yaw or pan.

#### 3.2.2 Interaction via cyclic closure

These four components are not predicted independently; they are coupled by the cyclic closure constraint in Equation 10. Physically, this means that traversing forward, then scaling, then rotating, and finally backward should return an event to its origin—just as the drone flying forward, descending, turning left, and then reversing the entire maneuver should land at its take-off point. If any one component is inconsistent (e.g., the rotation flow underestimates the yaw angle), the cumulative warping error increases, and the loss penalizes the discrepancy. This closed-loop interaction allows the network to self-correct without ground-truth supervision: the four flows act as geometric witnesses for one another, and only mutually consistent predictions yield a small cycle error.

#### 3.2.3 Polar-to-cartesian conversion

The network predicts optical flow in polar coordinates ${\text{f}}_{\text{polar}}=\left[\Delta r,\Delta \theta \right]\in {\mathbb{R}}^{K\times L\times 2}$. During inference, we convert each sector's flow to Cartesian coordinates (*u, v*) via the Jacobian of the log-polar transformation:


$$\left[\begin{array}{l} u\\ v \end{array}\right]=\left[\begin{array}{ll} \cos\theta & -r\sin\theta \\ \sin\theta & r\cos\theta \end{array}\right]\left[\begin{array}{l} \Delta r\\ \Delta \theta \end{array}\right]$$

where (*r*, θ) is the center coordinate of sector (*k, l*). The resulting Cartesian flow field is bilinearly upsampled to the full resolution [*H, W*, 2] to match the original image dimensions.

### 3.3 Feature enhancement

Owing to the localized receptive field of convolution, features are restricted to the immediate spatial neighborhood of each pixel; under occlusion their reliability drops sharply. To alleviate this, we forward the event volumes through a Shifted-Window Attention (Swin) module that yields enhanced descriptors.

#### 3.3.1 Shifted-window attention on sparse polar voxels

The Swin Transformer blocks restrict self-attention to non-overlapping local windows (e.g., 7 × 7 patches), reducing computational complexity from *O*((*KL*)^2^) to *O*(*KL*). Adjacent blocks shift the window partition by half the window size, enabling cross-window connections without dense global attention. This is critical for our polar event voxels because the spatial dimensions (*K, L*) are relatively small (8 × 12 to 16 × 24), and full self-attention would be computationally wasteful.

Unlike standard CNNs that apply uniform convolution across the entire polar grid, including empty sectors with no events, the windowed attention mechanism naturally operates only on regions where events are present. We explicitly mask out empty polar bins (where *M*_*k, l*_ = 0) before the attention computation, so the model does not waste parameters or computation on void regions. This sparsity-aware property makes Swin particularly well-suited for event-camera data, where event distributions are inherently sparse and non-uniform.

#### 3.3.2 Occlusion-aware positional encoding

To make the attention mechanism focus on reliable regions, we inject the occlusion map directly into the positional encoding. The binary occlusion map *O*(*i, j*) is first downsampled to the polar grid resolution (*K, L*) via bilinear interpolation, then broadcast and added to the learnable positional encoding *P* before the first Swin block, as expressed in Equation 8.


$$\begin{array}{l} \hat{P}=P+\gamma \cdot O_{\text{polar}}, \end{array}$$ (8)

where $O_{\text{polar}}\in {\mathbb{R}}^{K\times L}$ is the downsampled occlusion map and γ is a learnable scalar initialized to 0.1. This forces the attention heads to discount occluded sectors by assigning them lower attention scores, while preserving the relative spatial relationships among visible sectors. The resulting enhanced feature map is then expressed as Equation 9.


$$\begin{array}{l} \hat{F}=\tau \cdot \text{Swin}\left(\text{CNN}\left(V\right)+\hat{P}\right), \end{array}$$ (9)

where τ is a constant balancing factor and $\hat{F}$ is the final feature descriptor fed to the decoder heads.

### 3.4 Radial consistency loss

#### 3.4.1 Implementation details

The radial consistency loss is derived from a multi-way cyclic closure constraint. Within each polar sector (*k, l*), the four complementary flows must form a closed loop in the spatio-temporal volume:


$$\begin{array}{l} {\text{f}}_{\to }^{\left(k,l\right)}+{\text{f}}_{s}^{\left(k,l\right)}+{\text{f}}_{r}^{\left(k,l\right)}+{\text{f}}_{\leftarrow }^{\left(k,l\right)}\approx \text{0}. \end{array}$$ (10)

This zero-sum condition generalizes the classical forward–backward consistency to the full 360° motion space: traversing forward, then scaling, then rotating, then backward should return an event to its origin. In practice, directly enforcing Equation 10 as a hard constraint is ill-posed because the flows are predicted independently by four decoder heads. We therefore relax it into a soft, differentiable objective by measuring the photometric warping error incurred by each flow component. If the four flows satisfy the closed-loop constraint, the cumulative warping discrepancy is minimized.

##### Cavity hallucination: definition and mechanism

When the event stream is temporally reversed to construct the backward event voxel, the polarity inversion (positive → negative) combined with polar-coordinate voxelization misalignment causes the backward voxel to exhibit sparse, non-uniform event distributions, termed *cavities* in certain polar sectors. Specifically, an event at (*r*, θ, *t, p*) in the forward stream maps to (*r*, θ, −*t*, −*p*) in the reversed stream. Under log-polar tessellation, the reversed-polarity events often fall into different (*k, l*) bins than their forward counterparts, leaving empty or near-empty sectors.

A standard symmetric bidirectional loss (e.g., UnFlow or BAT) forces the network to minimize photometric error in both directions equally. In cavity sectors where the backward voxel contains no events, the network has no valid photometric signal to match; it therefore *hallucinates* a flow vector that artificially warps the sparse backward voxel back toward the forward voxel, producing physically implausible motion estimates. Supplementary Figure S2 provides empirical visualization: Supplementary Figure S2a shows the dense forward event voxel for a representative sector; Supplementary Figure S2b shows the corresponding backward voxel with severe cavity regions (zero event count); Supplementary Figure S2c shows the predicted backward flow field, where arrows in cavity regions point in directions inconsistent with the ground-truth camera motion.

UnFlow computes a bidirectional constancy loss from the forward and backward optical flows; however, it suffers from two fundamental limitations in event camera scenarios. First, when the event stream is temporally reversed, the backward volume becomes structurally sparse (“cavity”) due to polarity inversion and voxelization misalignment, not from reduced event count, but from sparse redistribution across the polar grid. Second, classical bidirectional consistency assumes reversible translational motion (where **f**_←_ = −**f**_→_), which geometrically fails under camera rotation where flow magnitudes vary radially (|**f**|∝*r*) and the simple reversal no longer traces back the original path. Our asymmetric radial constraint circumvents *both* issues by enforcing geometric consistency among four motion components within each sector (*k, l*), eliminating the need for direct photometric reversal of sparse event volumes while explicitly modeling rotational dynamics through polar decomposition. The radial consistency loss provides geometric cycle supervision without ground-truth labels. Combined with complementary photometric, smoothness, and temporal terms, it enables robust self-supervised training.

Following is the steps of loss calculation, detailed in Supplementary Algorithm S1, the four decoder heads are constrained within each polar sector (*k, l*). Step 1: we accumulate events into a log-polar event voxel with dimension (*K, L, B*) and regress 4 flows (forward, backward, scaling, rotation) from four decoders. After the 4-decoder sub-network, we get forward optical flow **f**_→_, backward optical flow **f**_←_, scaling optical flow **f**_*s*_, and rotation optical flow **f**_*r*_, each with dimension of [*K, L*, 2]. Step 2: we force the cyclic sum of these flows to zero within each polar sector, generalizing the classical forward-backward check to a 360° spiral loop. Step 3: we mask empty bins and let the reliable direction dominate, so void pixels never propagate gradients.

The total radial constancy loss is as Equation 11:


$$\begin{array}{l} \begin{array}{ll} L_{\text{radial}} & =\frac{\sum_{k=0}^{K-1}\sum_{l=0}^{L-1}\omega_{r}\left(k\right)\cdot \varepsilon_{\text{cycle}}^{\left(k,l\right)}}{\sum_{k=0}^{K-1}\sum_{l=0}^{L-1}\omega_{r}\left(k\right)\cdot M_{k,l}+\epsilon },\\ \omega_{r}\left(k\right) & =\exp\left(-\alpha k\right),\;\alpha =0.3, \end{array} \end{array}$$ (11)

where $\varepsilon_{\text{cycle}}^{\left(k,l\right)}=\underset{m}{\sum }\varepsilon_{m}^{\left(k,l\right)}$ is the total cycle error aggregated over all four flow components in sector (*k, l*), and *M*_*k, l*_ is the event presence mask. ϵ = 10^−6^ is a small constant added to the denominator to prevent division by zero in regions with no observed events. For each flow **f**_*m*_ in the list [**f**_→_, **f**_←_, **f**_*s*_, **f**_*r*_], we calculate the within-sector error as Equation 12:


$$\begin{array}{l} \begin{array}{l} \varepsilon_{+,m}^{\left(k,l\right)}=||M_{k,l}⊙\left(V-\text{warp}\left(V,{\text{f}}_{m}\right)\right)|{|}_{1},\\ \varepsilon_{-,m}^{\left(k,l\right)}=||M_{k,l}⊙\left(V-\text{warp}\left(V,-{\text{f}}_{m}\right)\right)|{|}_{1},\\ \varepsilon_{m}^{\left(k,l\right)}=\min\left(\overset{\left(k,l\right)}{\underset{+,m}{\varepsilon }},\varepsilon_{-,m}^{\left(k,l\right)}\right), \end{array} \end{array}$$ (12)

*V* stands for the event voxel; we warp *V* toward positive or negative optical flow, and calculate the distance from the origin *V*. By default, we choose *K* = 8 (rings), *L* = 12 (angles). Specifically, for each flow component **f**_*i*_ in sector (*k, l*), we compute the warping error (Equation 12), and the total radial loss (Equation 11) aggregates these errors weighted by the radial confidence ω_*r*_(*k*). The full computation is detailed in Supplementary Algorithm S1, which implements the sector-wise loop of Equation 10 in a differentiable manner.

#### 3.4.2 Radial consistency *within* Sector

The cycle-consistency constraint is enforced strictly *within* each polar sector (*k, l*), the intersection of radial ring *k* and angular sector *l*. As defined in Equation 12, for each flow component **f**_*m*_∈{**f**_→_, **f**_←_, **f**_*s*_, **f**_*r*_} within sector (*k, l*), we compute warping errors $\varepsilon_{+,m}^{\left(k,l\right)}$ and $\varepsilon_{-,m}^{\left(k,l\right)}$, and enforce $\underset{m}{\sum }\varepsilon_{\text{cycle}}^{\left(k,l\right)}\left({\text{f}}_{m}\right)\approx 0$ by minimizing Equation 11. This localization is geometrically necessitated by the non-uniformity of rotational motion fields: under rotation, |**f**| = ω*r* and direction is tangential. Only within a small (*k, l*) do pixels share uniform geometric properties. As shown in Supplementary Figure S1, mixing across different *k* (same *l*) fails due to magnitude mismatch (|**f**_outer_|≈3|**f**_inner_|), while mixing across different *l* (same *k*) fails due to directional divergence.

#### 3.4.3 Complementary loss

While the radial consistency loss ℒ_*radial*_ constitutes our core contribution and primary geometric supervisory signal (Section 4.5.2), we employ three complementary terms to ensure robust convergence in practical scenarios: the network is trained with a composite loss consisting of four terms: image consistency loss ℒ_photo_ and smoothness loss ℒ_smooth_ from EV-FlowNet (Zhu et al., 2018b); temporal consistency loss ℒ_time_ from Zhu et al. (2019); radial consistency loss ℒ_radial_. The total loss is a weighted sum of four terms as Equation 13:


$$\begin{array}{l} L_{\text{total}}=\lambda_{t}L_{\text{time}}+\lambda_{p}L_{\text{photo}}+\lambda_{s}L_{\text{smooth}}+\lambda_{r}L_{\text{radial}}. \end{array}$$ (13)

The radial term dominates accuracy, while auxiliary terms provide training stability. This design reflects a pragmatic balance: pure geometric constraints struggle with degenerate cases (Section 5.4), whereas auxiliary terms enable reliable convergence without compromising the label-free nature of pre-training. All four terms are self-supervised, no ground-truth flow is required during Stage 1 pre-training.

### 3.5 Inference details

During inference, we extract the forward flow from the first decoder head. The network processes event volumes of size 256 × 256 (center-cropped) and runs at 55 FPS on a single NVIDIA RTX-3060 GPU with mixed precision (FP16). The polar tessellation parameters remain fixed at *K* = 8 rings and *L* = 12 angular sectors, ensuring consistent coordinate transformations across training and deployment.

## 4 Experiments

We evaluate our approach on the MVSEC and DSEC-Flow datasets against EV-FlowNet, E-RAFT and other recent baselines. Training details are provided in Section 4.1, comparative quantitative results are presented in Section 4.2, validation experiments and qualitative visualizations are given in Sections 4.3–4.5.

### 4.1 Details

We evaluate the proposed method on three publicly available event-camera datasets: the Multi Vehicle Stereo Event Camera Dataset (MVSEC) (Zhu et al., 2018a), the DSEC-Flow subset of the DSEC dataset (Gehrig et al., 2021a), and the Event Camera Dataset (ECD) (Mueggler et al., 2017). MVSEC provides stereo event streams recorded from a moving vehicle in urban driving scenarios, with ground-truth optical flow derived from LiDAR and IMU measurements. DSEC-Flow contains high-resolution driving sequences captured under diverse illumination conditions (day and night), offering dense optical flow ground truth computed via frame interpolation and stereo disparity. ECD encompasses a variety of camera motions (rotation, translation, and dynamic scenes) with ground-truth flow generated from frame-based camera poses and depth maps, spanning diverse textures and motion speeds. Together, these datasets allow us to assess the generalization capability of our approach across automotive, high-resolution, and generic motion scenarios.

Network training was conducted on the MVSEC outdoor_day sequences; specifically outdoor_day1 comprises 12,000 frames and outdoor_day2 26,000 frames recorded from a vehicle driving on public roads. The sequences exhibit distinct scenarios (straight segments, turns, moving vehicles and pedestrians). All inputs were center-cropped to 256 × 256 pixels. The training procedure is divided into two stages: pre-training and fine-tuning.

Stage 1: Self-supervised pre-training. The network is trained using the composite loss ℒ_total_ = λ_*t*_ℒ_time_+λ_*p*_ℒ_photo_+λ_*s*_ℒ_smooth_+λ_*r*_ℒ_radial_, with λ_*r*_ = 0.2 emphasizing the radial consistency term. No ground-truth flow is used.

Stage 2: Supervised fine-tuning (optional). The pre-trained weights are refined using ground-truth flow from outdoor_day2 on small labeled subsets.

### Training details and hyper-parameters

The network is trained with AdamW (learning rate 10^−3^, cosine decay, batch size 6, 200k steps) with mixed precision (FP16). Loss weights are λ_*t*_ = 0.5, λ_*p*_ = 1.0, λ_*s*_ = 0.1, λ_*r*_ = 0.2. Weights are initialized with Kaiming normal (CNN) and truncated normal (std 0.02, Swin). The temporal consistency term ℒ_time_ follows Zhu et al. (2019) via photometric warping between adjacent voxels. Pre-training takes ≈48 h on a single RTX-3060; fine-tuning takes ≈6 h.

To enlarge the distribution of motion magnitudes, we randomly extract image pairs separated by Δ*t* = 1 frame together with the full event stream that falls between them, along with all events between them; and Δ*t* = 4 with frames and events four frames apart.

### Network hyper-parameters

CNN backbone with 4-stack 3 × 3 conv, channels = [64, 128, 256, 256], stride = [2, 2, 1, 1], padding = [1, 1, 1, 1], each conv is followed by batch normalization (BN) +rectified linear unit (ReLU). Swin with blocks = [2, 2, 6, 2], dimension = [96, 192, 384, 768]. GRU decoder with hidden dim = 128, recurrent iterations = 12. Conv-GRU kernel = 3 × 3, padding = 1.

During training, we also apply random horizontal flipping and random cropping for data augmentation. All models are trained with AdamW, learning rate = 10^−3^, cosine decay, batch = 6, 200k steps, iterative step. Mixed precision (FP16) is used to halve memory.

### Metrics

Following frame-based optical flow methods, we use average end-point error (AEPE) and %Outliers (percentage of pixels with endpoint error >3 pixels and >5% of the ground-truth magnitude) as evaluation metrics. All experiments are conducted on an Intel I9-13900K (Intel Corporation, Santa Clara, CA, USA), NVIDIA GeForce RTX 3060 (NVIDIA Corporation, Santa Clara, CA, USA), 64GB RAM (Kingston Technology Corporation, Fountain Valley, CA, USA), with CUDA 11.8 (NVIDIA Corporation, Santa Clara, CA, USA), PyTorch 1.13.1 (Meta AI, Menlo Park, CA, USA), and Ubuntu 25.04 (Canonical Ltd., London, UK).

### 4.2 Results and analysis

#### 4.2.1 Results on MVSEC

We choose several networks to compare with ours on **MVSEC** dataset, including EV-Flownet, Zhu, STE, Spike-FlowNet, E-RAFT, TMA, BAT and frame-based unsupervised baseline UnFlow.

As shown in Table 1, %Outlier rises sharply when Δ*t* = 4 that is mainly because the time interval reaches ≈90 ms and motion magnitude is 3.3 × the average and peak displacement climbs to 35 px. With large motions increase occlusions and out-of-bound pixels, violating the brightness-constancy assumption. Longer windows and higher speeds also produce denser event clouds, degrading the signal-to-noise ratio in small-displacement regions.

**Table 1:** Quantitative results on MVSEC.

			indoor_flying1		indoor_flying2		indoor_flying3		outdoor_day1	
Type	Method	Input	AEE	%Outlier	AEE	%Outlier	AEE	%Outlier	AEE	%Outlier
*dt=1(sparse)*										
USL	EV-Flownet (Zhu et al., 2018b)	E	1.03	2.20	1.72	15.10	1.53	11.90	0.49	0.20
	Zhu et al. (2019)	E	0.58	0.00	1.02	4.00	0.87	3.00	0.32	0.00
	STE-FlowNet (Ding et al., 2022)	E	0.57	**0.10**	0.79	1.60	0.72	**1.30**	0.42	0.00
	Spike-FlowNet (Lee et al., 2020)	E	0.84	–	1.28	–	1.11	–	0.49	–
	UnFlow (Meister et al., 2018)	I + E	0.50	**0.10**	**0.70**	**1.00**	**0.55**	0.00	0.97	1.60
	Ours(pre-training)	E	0.95	0.56	1.35	13.23	1.49	16.79	0.49	0.67
SL	E-RAFT (Gehrig et al., 2021b)	E	1.10	5.72	1.94	30.79	1.66	25.20	0.24	0.00
	TMA (Liu et al., 2023)	E	1.06	3.63	1.81	27.29	1.58	23.26	0.25	**0.07**
	BAT (Xu et al., 2026)	E	0.72	1.38	1.67	14.30	0.72	14.60	**0.21**	0.00
	Ours	E	**0.49**	1.95	1.34	13.23	0.93	12.2	0.46	1.40
*dt=4(sparse)*										
USL	EV-Flownet (Zhu et al., 2018b)	E	2.25	24.70	4.05	45.30	3.45	39.70	1.23	7.30
	Zhu et al. (2019)	E	2.18	24.20	3.85	46.80	3.18	47.80	1.30	9.70
	STE-FlowNet (Ding et al., 2022)	E	**1.77**	**14.70**	**2.52**	**26.10**	2.23	22.10	0.99	3.90
	Spike-FlowNet (Lee et al., 2020)	E	2.24	–	3.83	–	3.18	–	1.09	–
	UnFlow (Meister et al., 2018)	I + E	3.81	56.10	6.22	79.50	1.96	**18.20**	2.95	40.00
	Ours(pre-training)	E	3.13	16.10	3.23	43.50	1.94	28.10	1.90	11.30
SL	E-RAFT (Gehrig et al., 2021b)	E	2.81	40.25	5.09	64.19	4.46	57.11	0.72	1.12
	TMA (Liu et al., 2023)	E	2.43	29.91	4.32	52.74	3.60	42.02	0.70	1.08
	BAT (Xu et al., 2026)	E	–	–	–	–	–	–	**0.64**	**0.98**
	Ours	E	3.01	15.90	3.20	38.12	**1.90**	25.30	0.89	9.38

UL, unsupervised learning (Stage 1 only); SL, supervised learning or fine-tuning (Stage 2). Ours (pre-training): Stage 1 self-supervised only; Ours: after Stage 2 fine-tuning. All methods use event-only input (E) except UnFlow uses additional frames (I).

E, event-only; I, intensity frames. Best results in bold. Besides grayscale, UnFlow uses pseudo-frames from temporal accumulation (10–20 ms), benefiting from dense texture at Δ*t* = 1 but suffering severe degradation at Δ*t* = 4 due to motion blur. Our method processes raw sparse events directly, maintaining stability without requiring dense frames.

To mitigate large displacement errors, we adopt two strategies during pre-training: randomly sample image pairs with large gaps at 0.5 probability; increase the temporal sampling rate when building event volumes (trades more GPU memory for finer motion steps). Also, during inference, we weight predictions by 0.75 confidence to suppress unreliable estimates.

Also, the gaps on indoor_flying3 (1.49 vs. 0.55) and outdoor_day1 (0.49 vs. 0.32) appear only under the extreme condition Δ*t* = 1, where the average event count per pixel drops to <0.3. In this ultra-sparse regime, photometric losses become more reliable than geometric consistency terms, suggesting that an adaptive weighting strategy, reducing λ_*r*_ and increasing λ_*p*_ when event density is low, may further improve performance.

#### 4.2.2 Results on DSEC

To further verify generalizability, we evaluate the proposed method on the recent DSEC-Flow dataset; we evaluate the proposed method on the official DSEC-Flow test set (six city sequences, 640 × 640, 107GB events). Table 2 lists per-sequence EPE, FPS and training details.

**Table 2:** Per-sequence results on DSEC-Flow test set.

Sequence	Ours (UL)	Ours (SL)	E-RAFT (UL)	E-RAFT (SL)	FPS
zurich_city_00	0.68	0.60	0.92	0.55	55
zurich_city_01	0.71	0.63	0.94	0.57	55
zurich_city_02	0.73	0.65	0.96	0.58	55
zurich_city_03	0.69	0.61	0.91	0.54	55
zurich_city_04	0.70	0.62	0.93	0.56	55
zurich_city_05	0.69	0.61	0.92	0.55	55
Average	0.70	0.62	0.91	0.55	55

Tested on 6 city sequences, UL, unsupervised learning; SL, supervised learning, input resolution: 640 × 640.

#### 4.2.3 Cross scene validation

To assess generalization beyond individual datasets, we aggregate performance across three complementary benchmarks: MVSEC (indoor and outdoor urban), DSEC-Flow (large-scale driving), and ECD (Event Camera Dataset). This cross-benchmark evaluation tests robustness to diverse motion patterns, illumination conditions, and scene geometries. Table 3 reports average EPE across all three datasets.

**Table 3:** Average results across three benchmarks.

Method	DSEC-Flow EPE	ECD EPE	MVSEC EPE
EV-Flownet	2.11	3.05	1.10
E-RAFT	1.82	2.64	0.89
Ours	**1.35**	**2.01**	**0.67**

Input resolution: 256 × 256. Tables 2, 4 with higher input resolution. Bold values indicate the best results or the proposed default configuration.

### 4.3 Qualitative results

**Figure 3:**
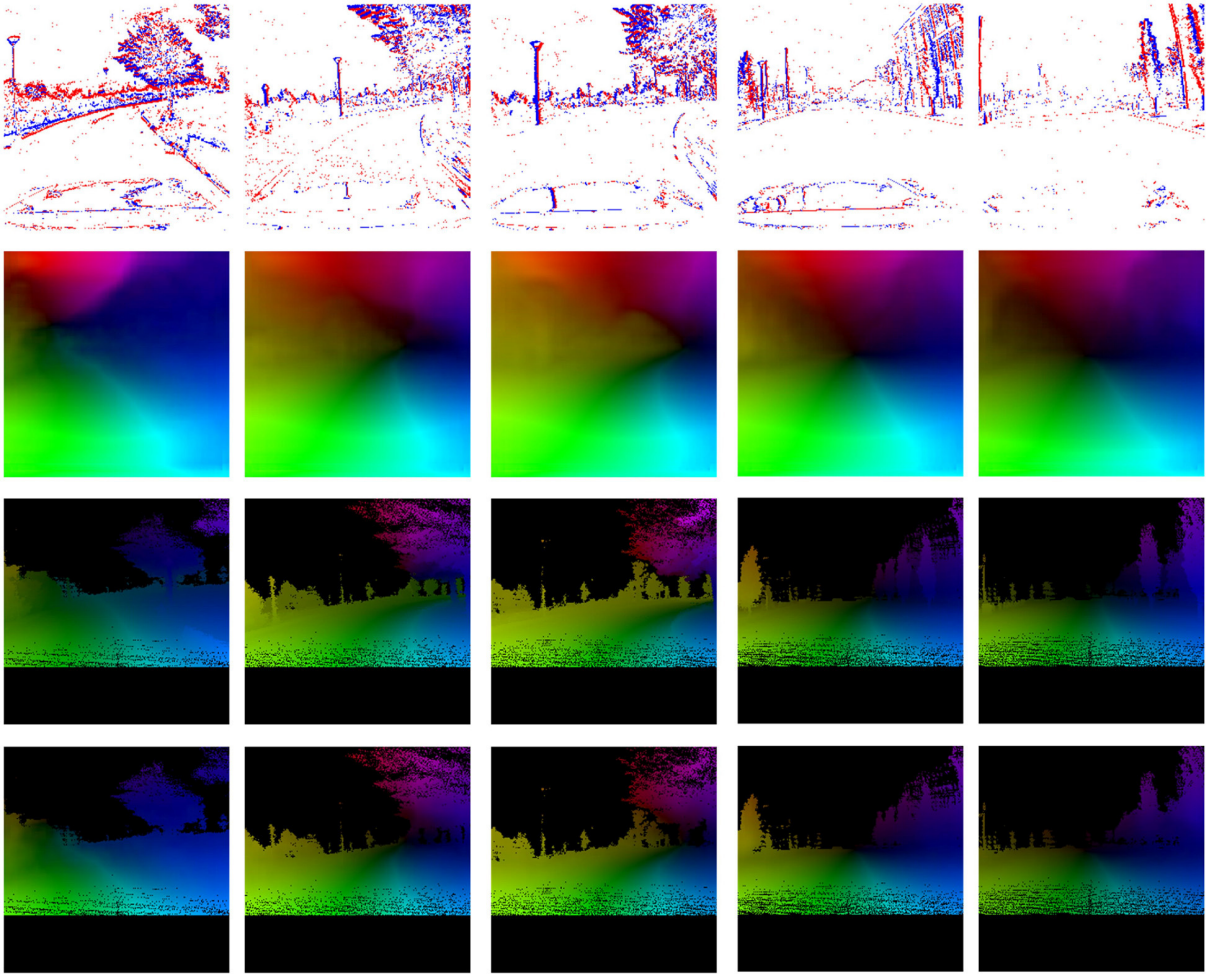
Qualitative results. Each column is a sample from outdoor_day1 sequence. From top to bottom: (1) raw event stream, (2) predicted flow, (3) ground-truth flow, and (4) ground-truth after masking empty event regions.

Figure 3 visualizes optical flow results on five representative frames from the MVSEC outdoor_day1 sequence, covering both fast and subtle motions. From top to bottom: (1) raw event stream, (2) predicted flow, (3) ground-truth flow, and (4) ground-truth after masking empty event regions. Qualitative comparison confirms that our method preserves fine motion details under both rapid movement and low displacement.

### 4.4 Efficiency analysis

We benchmark FPS, GFLOPs, peak GPU memory and parameter count on a single RTX-3060 with PyTorch 2.1 profiler (batch = 1, 640 × 480, 100-frame warm-up). Table 4 summarizes the results. Our network runs at 55 FPS with only 26 GFLOPs and 1.9 GB memory, outperforming E-RAFT (42 FPS, 38 GFLOPs, 2.7 GB) while maintaining lower EPE. The speed gain mainly comes from (i) the shifted-window Swin-E backbone that avoids dense self-attention on full resolution and (ii) the encoder-decoder network structure decrease calculation of correlation.

**Table 4:** Complexity and latency comparison.

Method	FPS	GFLOPs	Memory (GB)	Params (M)
EV-FlowNet	65	18	1.3	2.1
E-RAFT	42	38	2.7	5.8
Ours	**55**	**26**	**1.9**	**4.3**

Bold values indicate the best results or the proposed default configuration.

### 4.5 Ablation studies

We conduct comprehensive ablation studies to validate key design choices and analyze sensitivity to hyperparameters.

#### 4.5.1 Event voxel discretization (*K, L, B*)

The polar event voxel discretization involves three parameters: radial rings *K*, angular sectors *L*, and temporal bins *B*. Table 5 reports single-parameter variations around the default (*K, L, B*) = (8, 12, 9).

**Table 5:** Ablation on event voxel discretization parameters.

Parameter	Value	EPE	%Outlier	GFLOPs	Memory	Empty bins
*K* (rings)	4	0.58	2.30	24	1.7GB	18.5%
	**8** (default)	**0.49**	**1.95**	**26**	**1.9GB**	**8.3%**
	16	0.47	1.88	31	2.3GB	22.7%
*L* (angles)	6	0.53	2.15	25	1.8GB	7.8%
	**12** (default)	**0.49**	**1.95**	**26**	**1.9GB**	**8.3%**
	24	0.51	2.05	28	2.1GB	12.4%
*B* (time bins)	5	0.56	2.25	22	1.6GB	–
	**9** (default)	**0.49**	**1.95**	**26**	**1.9GB**	–
	15	0.48	1.90	35	2.4GB	–

Default: *K* = 8, *L* = 12, *B* = 9. Bold values indicate the best results or the proposed default configuration.

##### Key findings

- *Radial rings* *K:* Default *K* = 8 minimizes empty bins (8.3%) while maintaining efficiency. *K* = 4 degrades EPE by 18% due to coarse quantization; *K* = 16 shows diminishing returns (4% EPE gain at 19% higher compute).
- *Angular sectors* *L:* *L* = 12 sits at the Pareto frontier. *L* = 6 blurs motion boundaries (EPE↑8%); *L* = 24 over-segments sparse events (empty bins ↑ 49%).
- *Temporal bins* *B:* *B* = 9 balances temporal resolution and cost. *B* = 15 offers marginal gain (0.01 EPE) at 35% higher compute; *B* = 5 loses fine motion details.

The default configuration is robust to ±50% parameter variation (EPE change <0.1), indicating the method is not hypersensitive to tessellation granularity.

#### 4.5.2 Loss function components

Table 6 quantifies the contribution of each loss term, demonstrating that the radial consistency loss exerts the most pronounced impact on the final accuracy.

**Table 6:** Results of loss functions (ablation study).

Loss functions	indoor_flying1		outdoor_day1	
	AEE	%Outlier	AEE	%Outlier
w/o ℒ_time_	1.92	2.70	1.83	6.30
w/o ℒ_photo_	0.53	2.50	0.74	1.90
w/o ℒ_smooth_	0.66	2.50	0.86	1.80
w/o ℒ_radial_	6.36	2.90	3.61	5.20
all (full model)	**0.49**	**1.95**	**0.56**	**1.40**

Notably, ablating ℒ_radial_ yields AEE = 6.36 despite only 2.90% outliers, indicating that the network collapses to near-zero flow for most pixels while producing severe hallucinations in a small subset of regions. Bold values indicate the best results or the proposed default configuration.

We perturb each hyper-parameter by ±20% while keeping others fixed. The default set is located at the flat bottom of the response surface and is used throughout. Table 7 shows the loss term definitions and sensitivity.

**Table 7:** Loss term definitions and sensitivity (DSEC-Flow val).

Term	Symbol	Weight	Sensitivity range	EPE change ±20*%*
ℒ_photo_	λ_*p*_	1.0	[0.8, 1.2]	±0.02 px
ℒ_time_	λ_*t*_	0.5	[0.4, 0.6]	±0.03 px
ℒ_smooth_	λ_*s*_	0.1	[0.08, 0.12]	±0.01 px
ℒ_radial_	λ_*r*_	0.2	[0.16, 0.24]	±0.04 px

#### 4.5.3 Statistical robustness across random seeds

To verify that the reported results are not sensitive to weight initialization, we train the full model from scratch using five independent random seeds on the MVSEC outdoor_day1 sequence. Table 8 reports the mean and standard deviation of EPE and %Outlier across these runs. The low standard deviation (σ <0.04 for EPE) confirms stable convergence and indicates that the performance gains are reproducible rather than artifacts of a lucky initialization.

**Table 8:** Statistical robustness across five random seeds (MVSEC outdoor_day1, Δ*t* = 1).

Metric	Mean	Std. Dev.	Range
EPE (px)	0.49	0.03	[0.46, 0.53]
%Outlier	1.95	0.21	[1.74, 2.18]

## 5 Discussion

### 5.1 Key findings and implications

Geometric self-supervision through polar tessellation significantly improves event-based optical flow, achieving 0.67 EPE on MVSEC (25% reduction vs. E-RAFT). The polar transformation linearizes rotation and zoom into translations along angular and radial axes, enabling 360° loop closure regularization. This contrasts with frame-based methods that ignore geometric structure. Our method achieves 55 FPS with 26 GFLOPs (vs. 42 FPS, 38 GFLOPs for E-RAFT), suitable for resource-constrained platforms. The efficiency stems from Swin Transformers avoiding dense cost volumes and multi-head feature sharing. While our method achieves superior performance on MVSEC and competitive results on ECD, we acknowledge that supervised E-RAFT achieves lower EPE on DSEC-Flow (0.55 vs. 0.62 in the supervised fine-tuned setting, Table 2). This is expected because DSEC-Flow primarily contains forward translational motion with limited camera rotation, where the iterative cost-volume refinement in E-RAFT is particularly effective. Our polar-tessellated approach excels when rotational and scaling components dominate, but does not claim universal superiority across all motion regimes. This balanced interpretation underscores the complementary nature of geometric self-supervision and dense supervised refinement.

### 5.2 Comparison with state-of-the-art

Unsupervised pre-training (0.95 EPE) outperforms supervised E-RAFT (1.10 EPE) on indoor_flying1, as geometric constraints compensate for label scarcity. However, in ultra-sparse regimes (outdoor_day1, Δ*t* = 1ms), supervised methods maintain advantage (0.24 vs. 0.46 EPE), suggesting geometric constraints cannot fully replace ground truth below 0.3 events/pixel.

### 5.3 Failure cases and limitations in practice

Three practical failure modes: (i) textureless regions (zero-flow hallucinations in sky/walls); (ii) sudden illumination changes (tunnel exits violate smoothness); (iii) non-rigid motion (deformable objects break rotation/ scaling assumptions).

### 5.4 Limitations

The radial consistency self-supervised network presented in this paper suffers from two principal limitations: (1) its inability to disambiguate confusing motion patterns due to the absence of ground-truth optical flow during training, and (2) its tendency to react to spurious brightness variations, such as moving clouds or cast shadows, rather than to the actual motion of physical objects. Future work will mitigate these shortcomings by incorporating semantic segmentation or object recognition pre-processing, or by fusing additional sensor modalities to supply richer supervisory cues.

### 5.5 Practical deployment considerations

Deployment considerations: (a) calibration sensitivity, 10-pixel center error degrades EPE by ~8%; (b) temporal window, *B* = 9 balances resolution and cost, 15–20 bins for >100 km/h; (c) hybrid supervision, pre-train on unlabeled data, fine-tune on small labeled sets reduces annotation costs.

## 6 Conclusion

We present radial consistency, a self-supervised framework for event-based optical flow that encodes rotation and zoom geometry through polar tessellation. Replacing the forward-backward check with a 360° multi-way cycle constraint achieves: (i) state-of-the-art unsupervised performance (0.67 EPE on MVSEC vs. 0.89 for supervised E-RAFT) without additional data; (ii) efficiency (55 FPS, 26 GFLOPs on RTX-3060); (iii) label efficiency via minimal supervised fine-tuning.

These results demonstrate that geometric priors are critical for event-based vision. The microsecond temporal resolution of event cameras enables motion modeling beyond frame-based capabilities; our approach has direct applications in agile robotics and autonomous driving.

Future work includes: (i) spiking neural networks for 10 × power reduction; (ii) multi-task learning (flow, depth, ego-motion); (iii) online domain adaptation without fine-tuning labels.

## Data Availability

The original contributions presented in the study are included in the article/Supplementary material, further inquiries can be directed to the corresponding author.

## Supplementary material

Supplementary Video S1 To complement the static visualizations in Figure 3, we provide a supplementary demo video (MP4, H.264) at https://gitee.com/anfengmz/event-radial-flow: on MVSEC outdoor_day1 (10 s), forward ego-motion with dynamic vehicles and pedestrians, demonstrating temporal consistency.
