# Supplementary material for: Multi-way radial consistency pre-training for event based optical flow
Source: Front Neurorobot. 2026 Jul 16;20:1884626. doi: 10.3389/fnbot.2026.1884626 (PMC13422378; doi:10.3389/fnbot.2026.1884626)
Supplement: Supplementary Video S1 — To complement the static visualizations in Figure 3, we provide a supplementary demo video (MP4, H.264) at https://gitee.com/anfengmz/event-radial-flow: on MVSEC outdoor_day1 (10 s), forward ego-motion with dynamic vehicles and pedestrians, demonstrating temporal consistency. [file Data_Sheet_1.pdf]

frontiers

Supplementary Material

1 GEOMETRIC ANALYSIS OF SECTOR-WISE CONSTRAINT

Equation (10) in the main text enforces cycle consistency within each polar sector (k, l), rather than across the entire angular sector or radial ring. Here we provide geometric intuition for this design choice.

Under camera rotation, optical flow in polar coordinates exhibits two key properties: (i) flow direction is tangential (along θ), and (ii) flow magnitude scales with radius ($|\mathbf{f}| = \omega r$). Consequently, pixels at different radii or angles possess incompatible motion geometries that cannot be naively summed.

Supplementary Figure S1 illustrates three scenarios: (a) valid constraint within the same (k, l) where motion characteristics are consistent; (b) invalid mixing across radial rings (same l, different k) where magnitudes differ by $\sim 3\times$; (c) invalid mixing across angular sectors (same k, different l) where tangential directions diverge. Panel (d) abstracts the valid case, showing how four complementary flows form a closed loop within each sector.

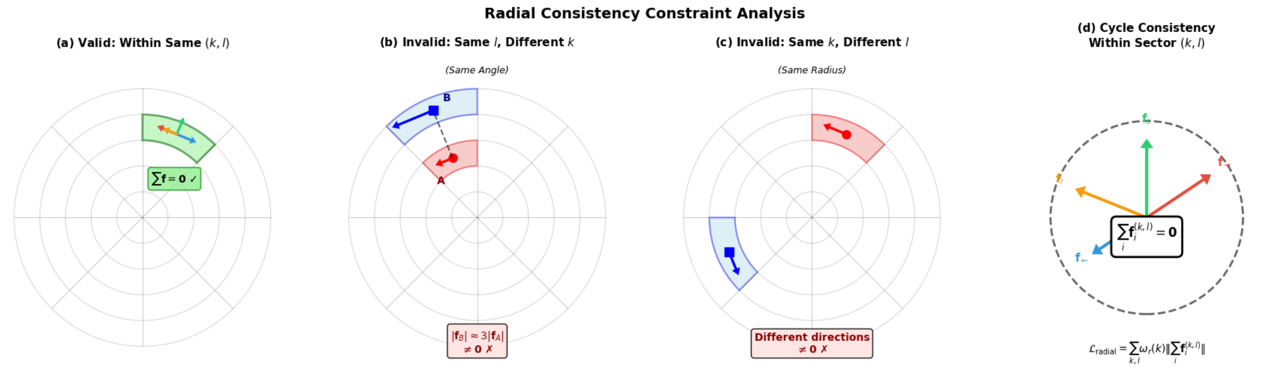

Figure S1: **Why radial consistency requires constraint within same (k, l) sector.**

(a) Valid case: four complementary flows within the same polar sector (k, l) sum to zero (green). (b) Invalid: same angular sector l but different radial rings k (A: inner, B: outer); motion magnitudes differ by $\sim 3\times$ due to $|\mathbf{f}| \propto r$. (c) Invalid: same radial ring k but different angular sectors l; tangential directions differ (90° apart). (d) Abstract illustration: within each sector (k, l), four flows ($\mathbf{f}_{\rightarrow}$, $\mathbf{f}_{\leftarrow}$, $\mathbf{f}_s$, $\mathbf{f}_r$) form a closed loop enforcing $\sum_i \mathbf{f}_i^{(k,l)} = \mathbf{0}$, aggregated by the radial consistency loss $\mathcal{L}_{\text{radial}}$.

2 RADIAL CONSISTENCY LOSS COMPUTATION

The radial consistency loss enforces geometric closure of SE(2) motion within each local polar sector (k, l). Unlike classical bidirectional methods that only verify forward-backward reversibility (assuming pure translation), our approach decomposes motion into four complementary components: temporal forward/backward flows ($\mathbf{f}_{\rightarrow}$, $\mathbf{f}_{\leftarrow}$), radial scaling ($\mathbf{f}_s$), and angular rotation ($\mathbf{f}_r$).

The computation proceeds in three stages: (i) estimating four flow fields from the shared encoder; (ii) computing warping errors independently within each non-empty polar sector (k, l), where we enforce that the cyclic sum of complementary flows approaches zero; and (iii) aggregating errors across all sectors with exponentially decaying radial weights $\omega_r(k)$ to account for varying event density.

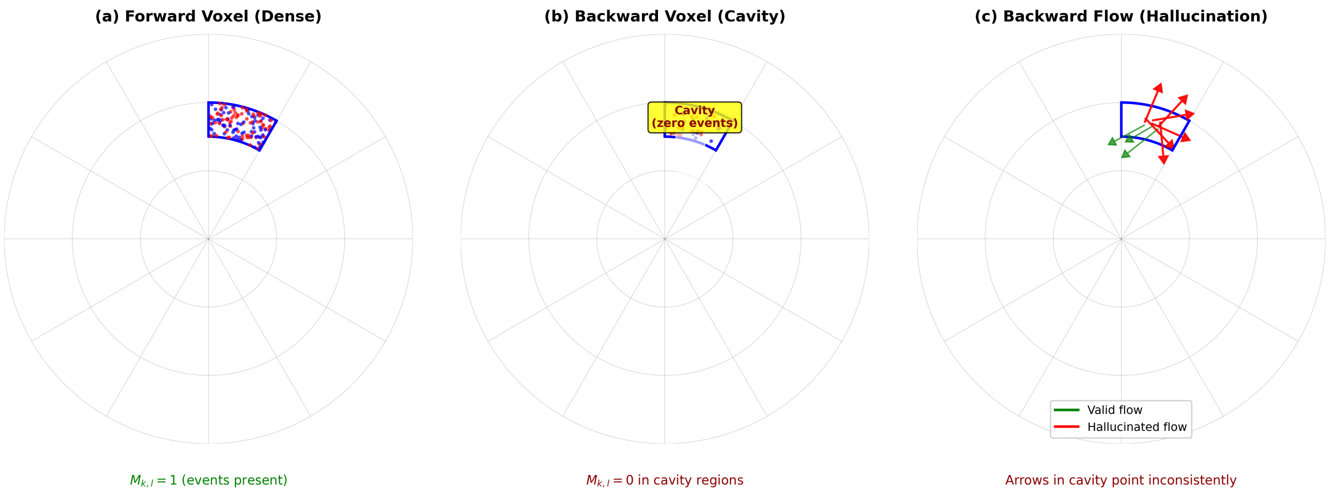

Figure S2: **Empirical evidence of cavity hallucination.** (a) Dense forward event voxel for a representative polar sector (k, l); events (positive in red, negative in blue) are densely distributed throughout the sector ($M_{k,l} = 1$). (b) Corresponding backward voxel after temporal reversal; polarity inversion and polar-coordinate redistribution leave severe *cavity* regions (highlighted in yellow) with zero event count ($M_{k,l} = 0$). (c) Predicted backward flow field; arrows in non-cavity regions (green) follow the physically correct ego-motion direction, whereas arrows inside cavity regions (red) point in random inconsistent directions, demonstrating that symmetric bidirectional losses *hallucinate* implausible flow when no valid photometric signal is present.

Algorithm 1 details this procedure. Key distinctions from prior work include: (a) sector-wise masking $M_{k,l}$ that excludes empty voxels from gradient computation; (b) asymmetric weighting that allows reliable directions to dominate; and (c) the four-way cycle constraint generalizing classical two-way consistency.

Algorithm 1 Radial Consistency Loss Computation

Require: Polar event voxels V_f, V_b (forward/backward), tessellation parameters (K, L), weights $\lambda_t, \lambda_p, \lambda_s, \lambda_r$
Ensure: Total loss $\mathcal{L}_{\text{total}}$

// Step 1: Estimate four complementary flows
$\mathbf{f}_{\rightarrow}, \mathbf{f}_{\leftarrow}, \mathbf{f}_s, \mathbf{f}_r \leftarrow \text{Network}(V_f, V_b)$
$\mathcal{F} \leftarrow \{\mathbf{f}_{\rightarrow}, \mathbf{f}_{\leftarrow}, \mathbf{f}_s, \mathbf{f}_r\}$
// Step 2: Compute auxiliary losses (photometric, smoothness, temporal)
$\mathcal{L}_{\text{photo}} \leftarrow \text{PhotometricLoss}(V_f, \mathbf{f}_{\rightarrow})$
$\mathcal{L}_{\text{smooth}} \leftarrow \text{SmoothnessLoss}(\mathbf{f}_{\rightarrow})$
$\mathcal{L}_{\text{time}} \leftarrow \text{TemporalConsistency}(V_f, V_b)$
// Step 3: Compute Radial Consistency Loss
$\mathcal{L}_{\text{radial}} \leftarrow 0, W \leftarrow 0$
for $k = 0$ to $K - 1$ **do**
for $l = 0$ to $L - 1$ **do**
// Check if sector (k, l) contains events
$M_{k,l} \leftarrow \mathbb{I}(\text{count}(V_f^{(k,l)}) > 0)$
if $M_{k,l} = 1$ **then**
// Compute cycle error for each flow component within sector (k, l)
$\varepsilon_{\text{cycle}}^{(k,l)} \leftarrow 0$
for $\mathbf{f}_i \in \mathcal{F}$ **do**
// Forward and backward warping errors (Eq. 8)
$\varepsilon_+ \leftarrow \|V_f^{(k,l)} - \text{warp}(V_f^{(k,l)}, \mathbf{f}_i)\|_1$
$\varepsilon_- \leftarrow \|V_f^{(k,l)} - \text{warp}(V_f^{(k,l)}, -\mathbf{f}_i)\|_1$
$\varepsilon_{\text{cycle}}^{(k,l)} \leftarrow \varepsilon_{\text{cycle}}^{(k,l)} + \min(\varepsilon_+, \varepsilon_-)$
end for
// Accumulate weighted error (Eq. 7)
$\omega_r(k) \leftarrow \exp(-\alpha k)$ {Radial confidence decay}
$\mathcal{L}_{\text{radial}} \leftarrow \mathcal{L}_{\text{radial}} + \omega_r(k) \cdot \varepsilon_{\text{cycle}}^{(k,l)}$
$W \leftarrow W + \omega_r(k)$
end if
end for
end for
$\mathcal{L}_{\text{radial}} \leftarrow \mathcal{L}_{\text{radial}}/W$ {Normalize}
// Step 4: Aggregate total loss (Eq. 9)
$\mathcal{L}_{\text{total}} \leftarrow \lambda_t \mathcal{L}_{\text{time}} + \lambda_p \mathcal{L}_{\text{photo}} + \lambda_s \mathcal{L}_{\text{smooth}} + \lambda_r \mathcal{L}_{\text{radial}}$
return $\mathcal{L}_{\text{total}}$
